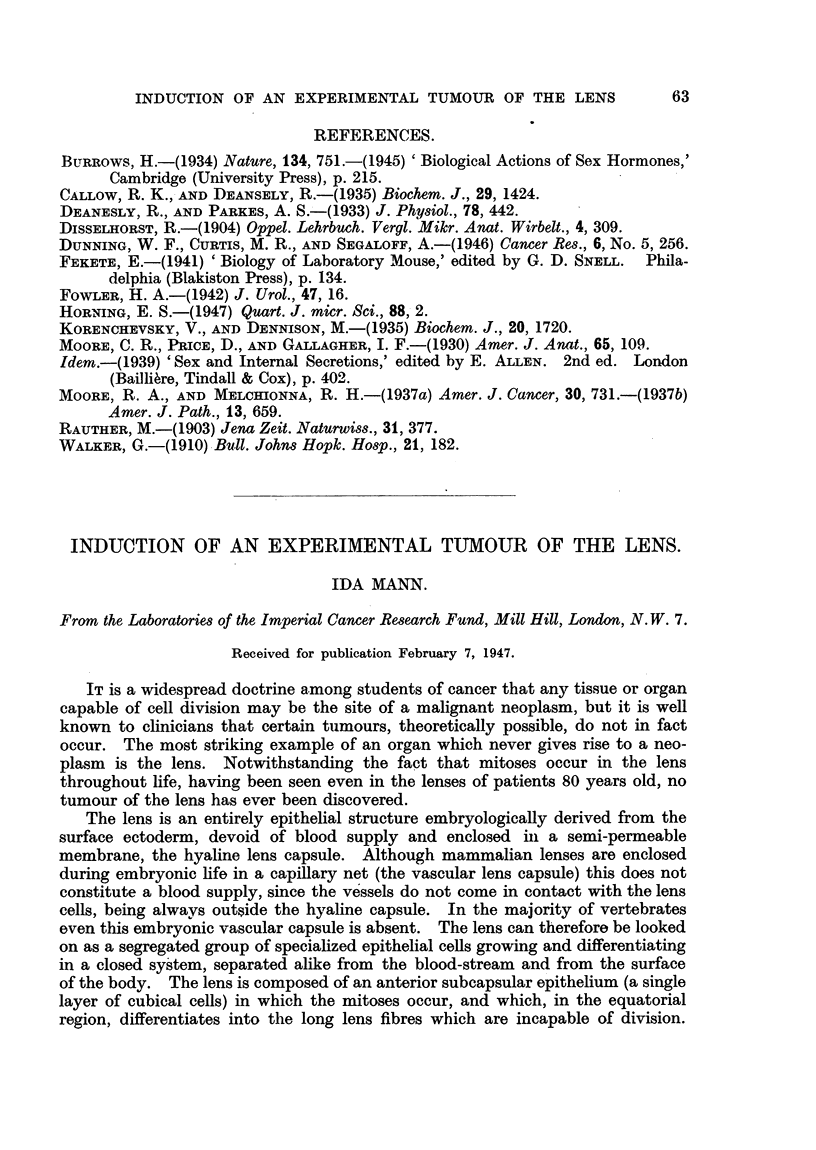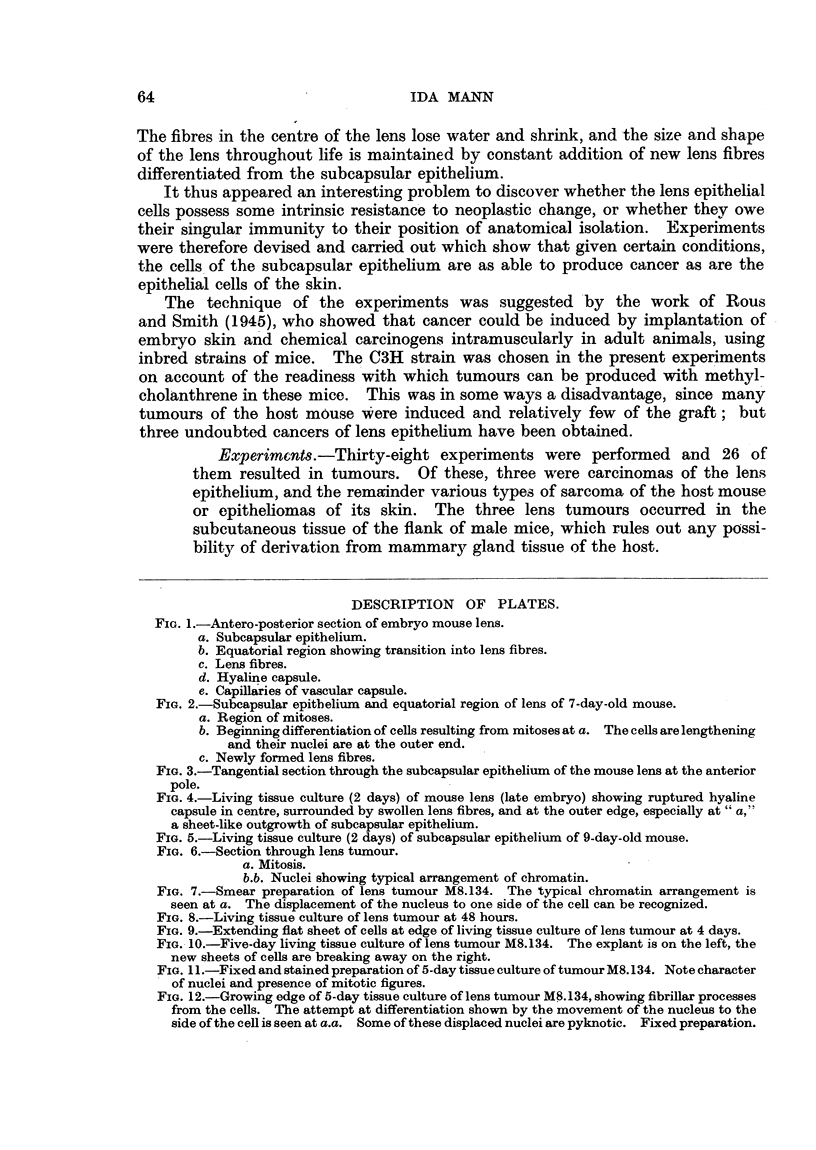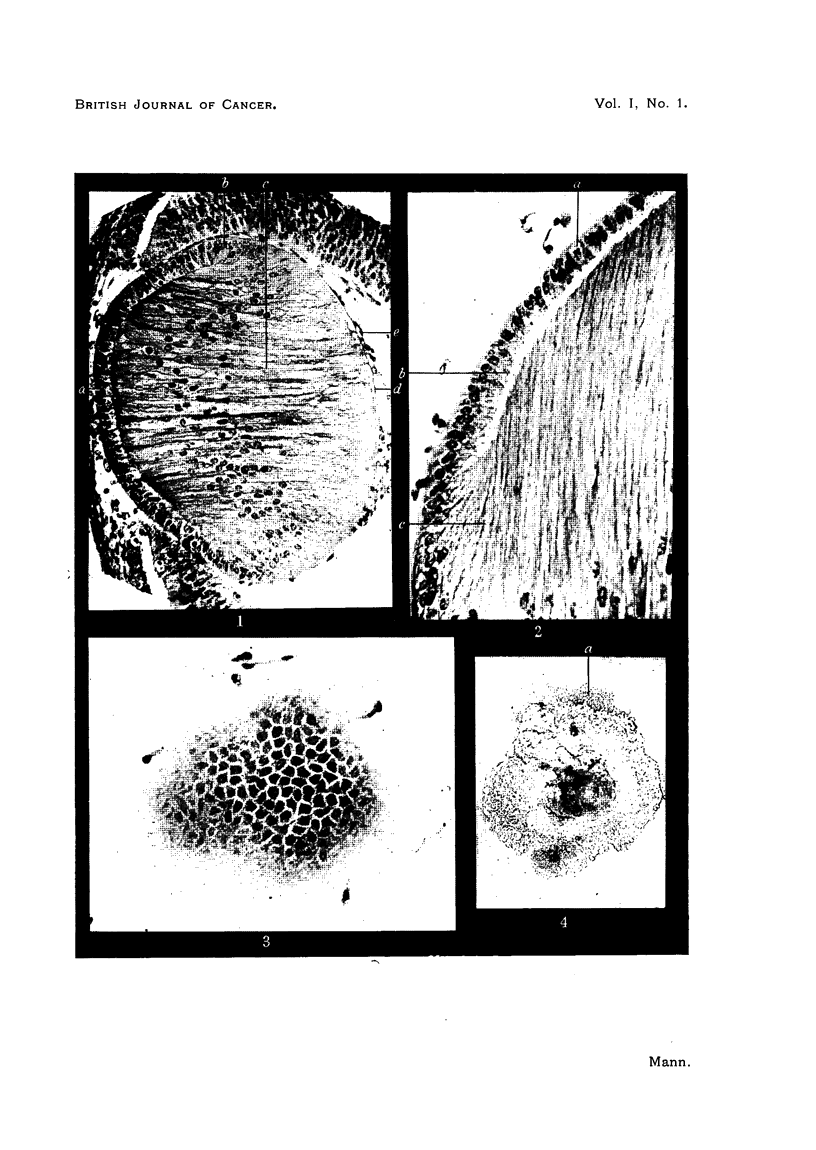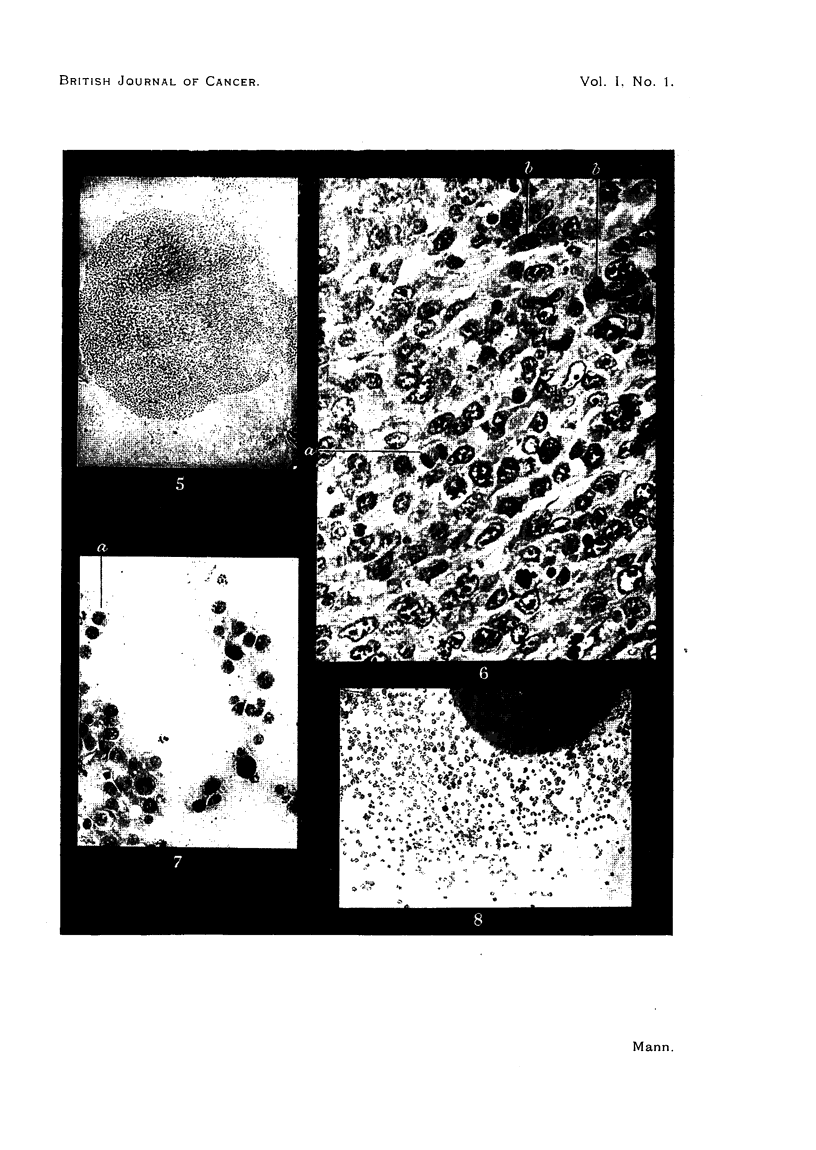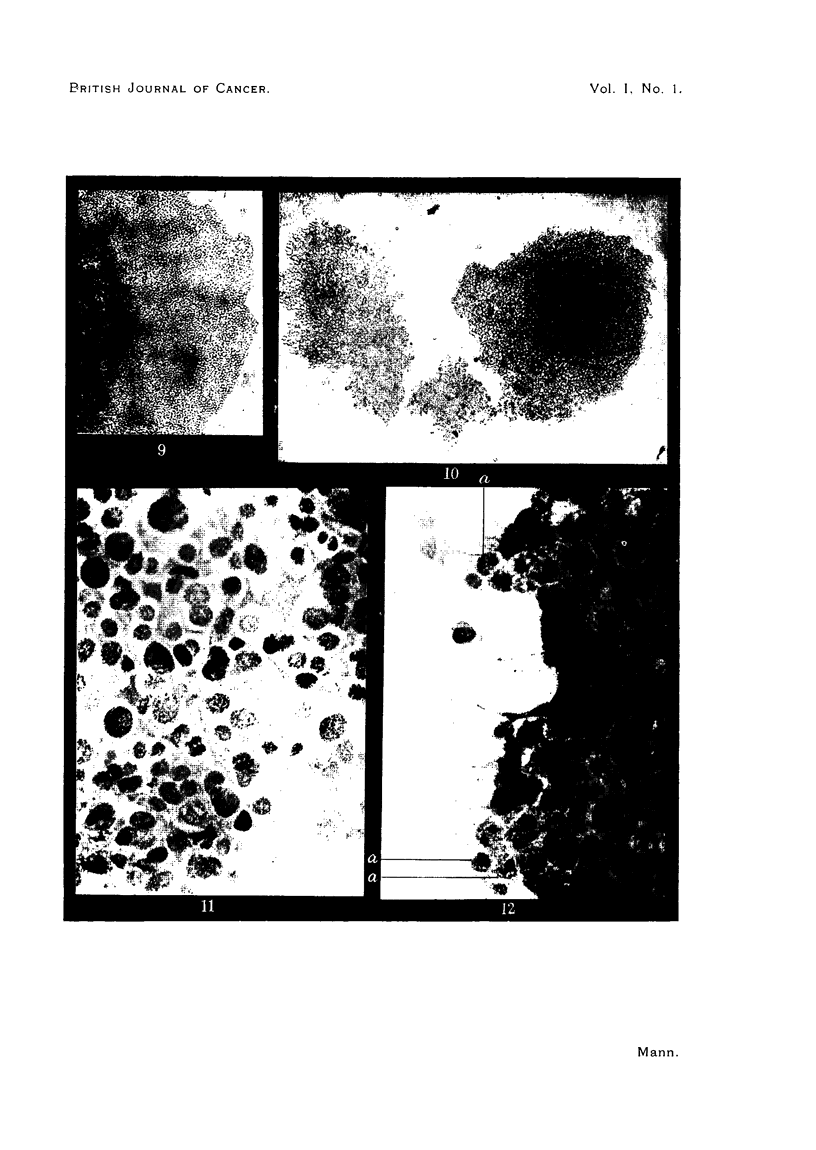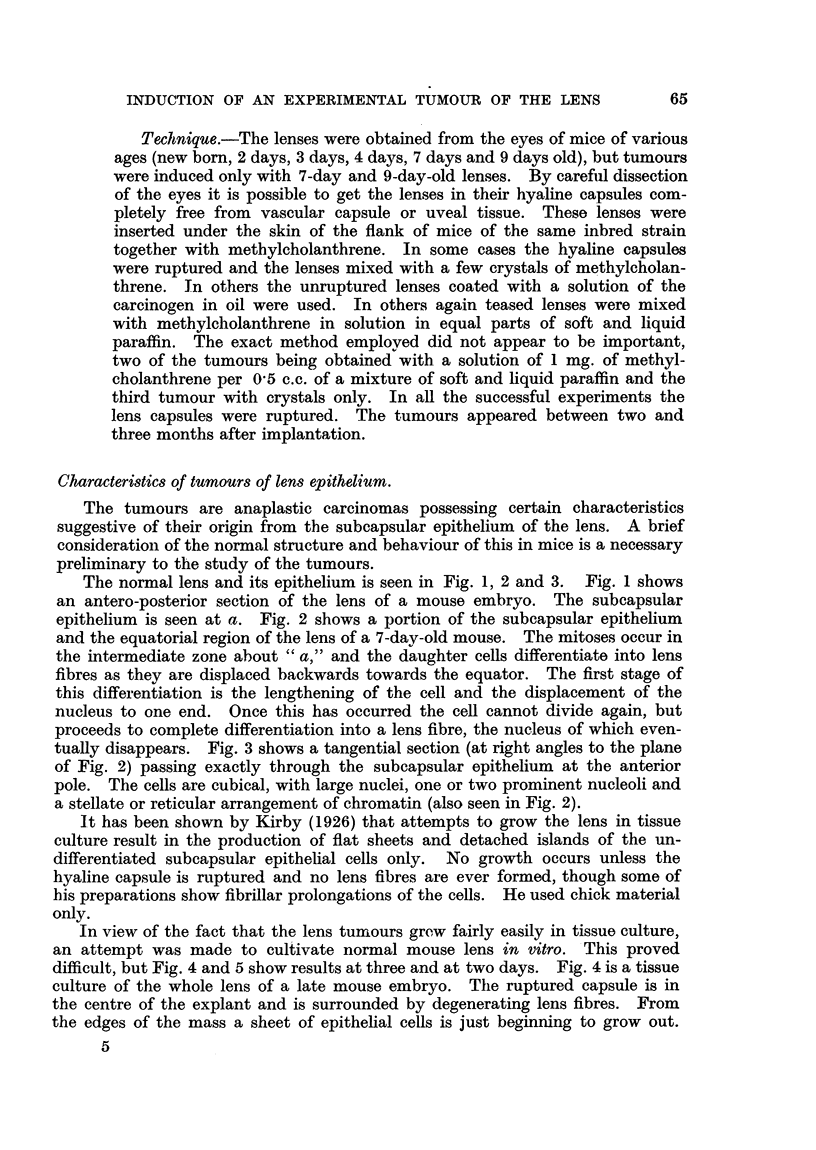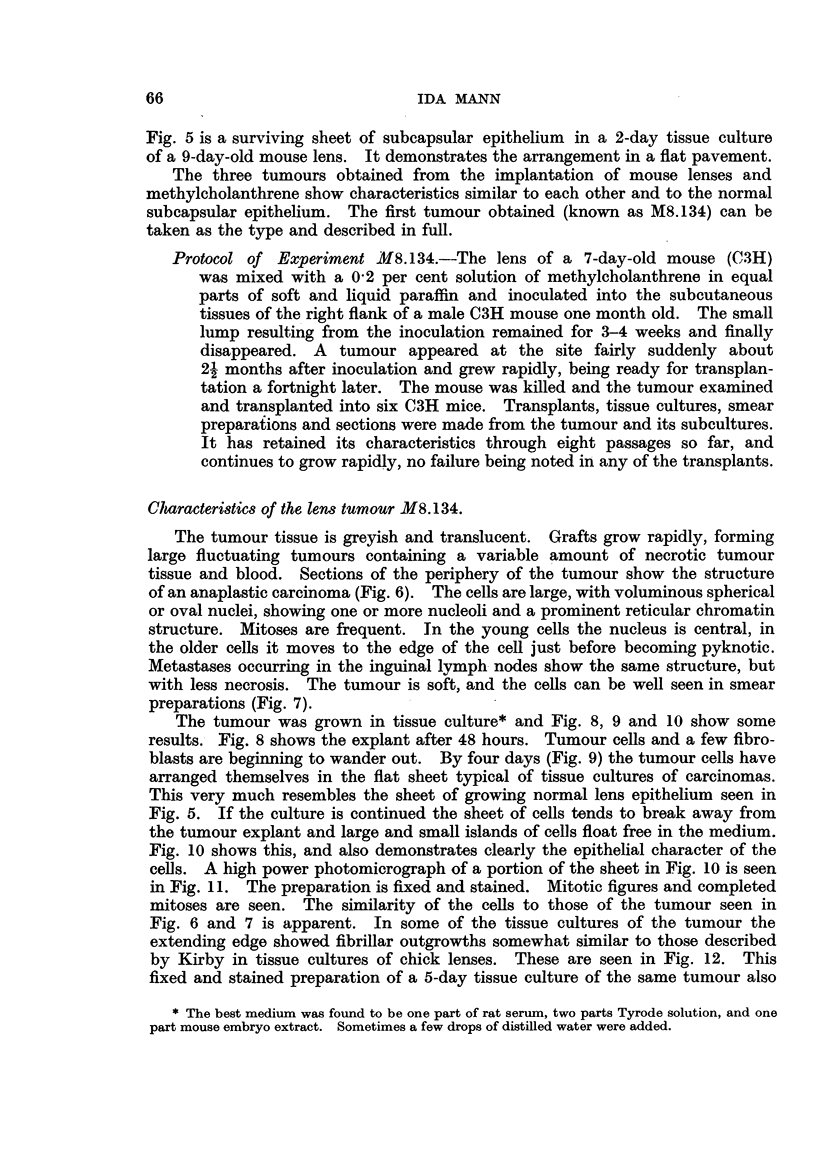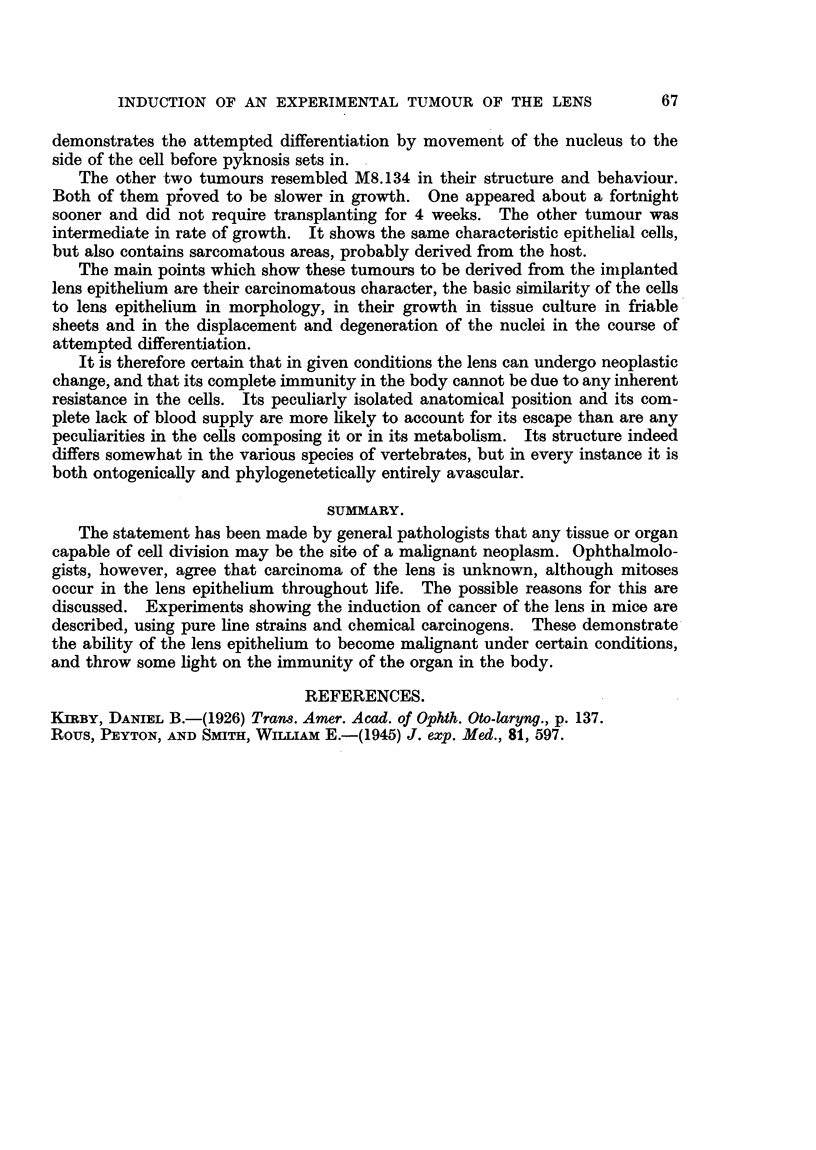# Induction of an Experimental Tumour of the Lens

**DOI:** 10.1038/bjc.1947.8

**Published:** 1947-03

**Authors:** Ida Mann

## Abstract

**Images:**


### INDUCTION OF AN EXPERIMENTAL TUMOUR OF THE LENS.

IDA MANN.

*From the Laboratories of the Imperial Cancer Research Fund, Mill Hill, London, N.W. 7.*

Received for publication February 7, 1947.

IT is a widespread doctrine among students of cancer that any tissue or organ capable of cell division may be the site of a malignant neoplasm, but it is well known to clinicians that certain tumours, theoretically possible, do not in fact occur. The most striking example of an organ which never gives rise to a neoplasm is the lens. Notwithstanding the fact that mitoses occur in the lens throughout life, having been seen even in the lenses of patients 80 years old, no tumour of the lens has ever been discovered.

The lens is an entirely epithelial structure embryologically derived from the surface ectoderm, devoid of blood supply and enclosed in a semi-permeable membrane, the hyaline lens capsule. Although mammalian lenses are enclosed during embryonic life in a capillary net (the vascular lens capsule) this does not constitute a blood supply, since the vessels do not come in contact with the lens cells, being always outside the hyaline capsule. In the majority of vertebrates even this embryonic vascular capsule is absent. The lens can therefore be looked on as a segregated group of specialized epithelial cells growing and differentiating in a closed system, separated alike from the blood-stream and from the surface of the body. The lens is composed of an anterior subcapsular epithelium (a single layer of cubical cells) in which the mitoses occur, and which, in the equatorial region, differentiates into the long lens fibres which are incapable of division.

The fibres in the centre of the lens lose water and shrink, and the size and shape of the lens throughout life is maintained by constant addition of new lens fibres differentiated from the subcapsular epithelium.

It thus appeared an interesting problem to discover whether the lens epithelial cells possess some intrinsic resistance to neoplastic change, or whether they owe their singular immunity to their position of anatomical isolation. Experiments were therefore devised and carried out which show that given certain conditions, the cells of the subcapsular epithelium are as able to produce cancer as are the epithelial cells of the skin.

The technique of the experiments was suggested by the work of Rous and Smith (1945), who showed that cancer could be induced by implantation of embryo skin and chemical carcinogens intramuscularly in adult animals, using inbred strains of mice. The C3H strain was chosen in the present experiments on account of the readiness with which tumours can be produced with methylcholanthrene in these mice. This was in some ways a disadvantage, since many tumours of the host mouse were induced and relatively few of the graft; but three undoubted cancers of lens epithelium have been obtained.

*Experiments.*—Thirty-eight experiments were performed and 26 of them resulted in tumours. Of these, three were carcinomas of the lens epithelium, and the remainder various types of sarcoma of the host mouse or epitheliomas of its skin. The three lens tumours occurred in the subcutaneous tissue of the flank of male mice, which rules out any possibility of derivation from mammary gland tissue of the host.

---

### DESCRIPTION OF PLATES.

FIG. 1.—Antero-posterior section of embryo mouse lens.
- *a.* Subcapsular epithelium.
- *b.* Equatorial region showing transition into lens fibres.
- *c.* Lens fibres.
- *d.* Hyaline capsule.
- *e.* Capillaries of vascular capsule.

FIG. 2.—Subcapsular epithelium and equatorial region of lens of 7-day-old mouse.
- *a.* Region of mitoses.
- *b.* Beginning differentiation of cells resulting from mitoses at *a.* The cells are lengthening and their nuclei are at the outer end.
- *c.* Newly formed lens fibres.

FIG. 3.—Tangential section through the subcapsular epithelium of the mouse lens at the anterior pole.

FIG. 4.—Living tissue culture (2 days) of mouse lens (late embryo) showing ruptured hyaline capsule in centre, surrounded by swollen lens fibres, and at the outer edge, especially at "*a*," a sheet-like outgrowth of subcapsular epithelium.

FIG. 5.—Living tissue culture (2 days) of subcapsular epithelium of 9-day-old mouse.

FIG. 6.—Section through lens tumour.
- *a.* Mitosis.
- *b.b.* Nuclei showing typical arrangement of chromatin.

FIG. 7.—Smear preparation of lens tumour M8.134. The typical chromatin arrangement is seen at *a.* The displacement of the nucleus to one side of the cell can be recognized.

FIG. 8.—Living tissue culture of lens tumour at 48 hours.

FIG. 9.—Extending flat sheet of cells at edge of living tissue culture of lens tumour at 4 days.

FIG. 10.—Five-day living tissue culture of lens tumour M8.134. The explant is on the left, the new sheets of cells are breaking away on the right.

FIG. 11.—Fixed and stained preparation of 5-day tissue culture of tumour M8.134. Note character of nuclei and presence of mitotic figures.

FIG. 12.—Growing edge of 5-day tissue culture of lens tumour M8.134, showing fibrillar processes from the cells. The attempt at differentiation shown by the movement of the nucleus to the side of the cell is seen at *a.a.* Some of these displaced nuclei are pyknotic. Fixed preparation.

Mann.

5

6

7

8

Mann.

9
10
11
12
a
a
a

*Technique.*—The lenses were obtained from the eyes of mice of various ages (new born, 2 days, 3 days, 4 days, 7 days and 9 days old), but tumours were induced only with 7-day and 9-day-old lenses. By careful dissection of the eyes it is possible to get the lenses in their hyaline capsules completely free from vascular capsule or uveal tissue. These lenses were inserted under the skin of the flank of mice of the same inbred strain together with methylcholanthrene. In some cases the hyaline capsules were ruptured and the lenses mixed with a few crystals of methylcholanthrene. In others the unruptured lenses coated with a solution of the carcinogen in oil were used. In others again teased lenses were mixed with methylcholanthrene in solution in equal parts of soft and liquid paraffin. The exact method employed did not appear to be important, two of the tumours being obtained with a solution of 1 mg. of methylcholanthrene per 0·5 c.c. of a mixture of soft and liquid paraffin and the third tumour with crystals only. In all the successful experiments the lens capsules were ruptured. The tumours appeared between two and three months after implantation.

*Characteristics of tumours of lens epithelium.*

The tumours are anaplastic carcinomas possessing certain characteristics suggestive of their origin from the subcapsular epithelium of the lens. A brief consideration of the normal structure and behaviour of this in mice is a necessary preliminary to the study of the tumours.

The normal lens and its epithelium is seen in Fig. 1, 2 and 3. Fig. 1 shows an antero-posterior section of the lens of a mouse embryo. The subcapsular epithelium is seen at *a*. Fig. 2 shows a portion of the subcapsular epithelium and the equatorial region of the lens of a 7-day-old mouse. The mitoses occur in the intermediate zone about "*a*," and the daughter cells differentiate into lens fibres as they are displaced backwards towards the equator. The first stage of this differentiation is the lengthening of the cell and the displacement of the nucleus to one end. Once this has occurred the cell cannot divide again, but proceeds to complete differentiation into a lens fibre, the nucleus of which eventually disappears. Fig. 3 shows a tangential section (at right angles to the plane of Fig. 2) passing exactly through the subcapsular epithelium at the anterior pole. The cells are cubical, with large nuclei, one or two prominent nucleoli and a stellate or reticular arrangement of chromatin (also seen in Fig. 2).

It has been shown by Kirby (1926) that attempts to grow the lens in tissue culture result in the production of flat sheets and detached islands of the undifferentiated subcapsular epithelial cells only. No growth occurs unless the hyaline capsule is ruptured and no lens fibres are ever formed, though some of his preparations show fibrillar prolongations of the cells. He used chick material only.

In view of the fact that the lens tumours grow fairly easily in tissue culture, an attempt was made to cultivate normal mouse lens *in vitro*. This proved difficult, but Fig. 4 and 5 show results at three and at two days. Fig. 4 is a tissue culture of the whole lens of a late mouse embryo. The ruptured capsule is in the centre of the explant and is surrounded by degenerating lens fibres. From the edges of the mass a sheet of epithelial cells is just beginning to grow out.

Fig. 5 is a surviving sheet of subcapsular epithelium in a 2-day tissue culture of a 9-day-old mouse lens. It demonstrates the arrangement in a flat pavement.

The three tumours obtained from the implantation of mouse lenses and methylcholanthrene show characteristics similar to each other and to the normal subcapsular epithelium. The first tumour obtained (known as M8.134) can be taken as the type and described in full.

*Protocol of Experiment M*8.134.—The lens of a 7-day-old mouse (C3H) was mixed with a 0·2 per cent solution of methylcholanthrene in equal parts of soft and liquid paraffin and inoculated into the subcutaneous tissues of the right flank of a male C3H mouse one month old. The small lump resulting from the inoculation remained for 3–4 weeks and finally disappeared. A tumour appeared at the site fairly suddenly about 2½ months after inoculation and grew rapidly, being ready for transplantation a fortnight later. The mouse was killed and the tumour examined and transplanted into six C3H mice. Transplants, tissue cultures, smear preparations and sections were made from the tumour and its subcultures. It has retained its characteristics through eight passages so far, and continues to grow rapidly, no failure being noted in any of the transplants.

*Characteristics of the lens tumour M*8.134.

The tumour tissue is greyish and translucent. Grafts grow rapidly, forming large fluctuating tumours containing a variable amount of necrotic tumour tissue and blood. Sections of the periphery of the tumour show the structure of an anaplastic carcinoma (Fig. 6). The cells are large, with voluminous spherical or oval nuclei, showing one or more nucleoli and a prominent reticular chromatin structure. Mitoses are frequent. In the young cells the nucleus is central, in the older cells it moves to the edge of the cell just before becoming pyknotic. Metastases occurring in the inguinal lymph nodes show the same structure, but with less necrosis. The tumour is soft, and the cells can be well seen in smear preparations (Fig. 7).

The tumour was grown in tissue culture* and Fig. 8, 9 and 10 show some results. Fig. 8 shows the explant after 48 hours. Tumour cells and a few fibroblasts are beginning to wander out. By four days (Fig. 9) the tumour cells have arranged themselves in the flat sheet typical of tissue cultures of carcinomas. This very much resembles the sheet of growing normal lens epithelium seen in Fig. 5. If the culture is continued the sheet of cells tends to break away from the tumour explant and large and small islands of cells float free in the medium. Fig. 10 shows this, and also demonstrates clearly the epithelial character of the cells. A high power photomicrograph of a portion of the sheet in Fig. 10 is seen in Fig. 11. The preparation is fixed and stained. Mitotic figures and completed mitoses are seen. The similarity of the cells to those of the tumour seen in Fig. 6 and 7 is apparent. In some of the tissue cultures of the tumour the extending edge showed fibrillar outgrowths somewhat similar to those described by Kirby in tissue cultures of chick lenses. These are seen in Fig. 12. This fixed and stained preparation of a 5-day tissue culture of the same tumour also

* The best medium was found to be one part of rat serum, two parts Tyrode solution, and one part mouse embryo extract. Sometimes a few drops of distilled water were added.

demonstrates the attempted differentiation by movement of the nucleus to the side of the cell before pyknosis sets in.

The other two tumours resembled M8.134 in their structure and behaviour. Both of them proved to be slower in growth. One appeared about a fortnight sooner and did not require transplanting for 4 weeks. The other tumour was intermediate in rate of growth. It shows the same characteristic epithelial cells, but also contains sarcomatous areas, probably derived from the host.

The main points which show these tumours to be derived from the implanted lens epithelium are their carcinomatous character, the basic similarity of the cells to lens epithelium in morphology, in their growth in tissue culture in friable sheets and in the displacement and degeneration of the nuclei in the course of attempted differentiation.

It is therefore certain that in given conditions the lens can undergo neoplastic change, and that its complete immunity in the body cannot be due to any inherent resistance in the cells. Its peculiarly isolated anatomical position and its complete lack of blood supply are more likely to account for its escape than are any peculiarities in the cells composing it or in its metabolism. Its structure indeed differs somewhat in the various species of vertebrates, but in every instance it is both ontogenically and phylogenetetically entirely avascular.

SUMMARY.

The statement has been made by general pathologists that any tissue or organ capable of cell division may be the site of a malignant neoplasm. Ophthalmologists, however, agree that carcinoma of the lens is unknown, although mitoses occur in the lens epithelium throughout life. The possible reasons for this are discussed. Experiments showing the induction of cancer of the lens in mice are described, using pure line strains and chemical carcinogens. These demonstrate the ability of the lens epithelium to become malignant under certain conditions, and throw some light on the immunity of the organ in the body.

### REFERENCES.

Kirby, Daniel B.—(1926) *Trans. Amer. Acad. of Ophth. Oto-laryng.*, p. 137.
Rous, Peyton, and Smith, William E.—(1945) *J. exp. Med.*, **81**, 597.